# Extracellular vesicle-coupled miRNA profiles in follicular fluid of cows with divergent post-calving metabolic status

**DOI:** 10.1038/s41598-019-49029-9

**Published:** 2019-09-06

**Authors:** Tsige Hailay, Michael Hoelker, Mikhael Poirier, Samuel Gebremedhn, Franca Rings, Mohammed Saeed-Zidane, Dessie Salilew-Wondim, Christina Dauben, Ernst Tholen, Christiane Neuhoff, Karl Schellander, Dawit Tesfaye

**Affiliations:** Institute of Animal Science, Department of Animal Breeding and Husbandry, University of Bonn, Bonn, Germany

**Keywords:** Functional genomics, Molecular medicine

## Abstract

Most high-yielding dairy cows enter a state of negative energy balance (NEB) during early lactation. This, in turn, results in changes in the level of various metabolites in the blood and follicular fluid microenvironment which contributes to disturbed fertility. Extracellular vesicles (EVs) are evolutionarily conserved communicasomes that transport cargo of miRNA, proteins and lipids. EV-coupled miRNAs have been reported in follicular fluid. However, the association between postpartum NEB and EV-coupled miRNA signatures in follicular fluid is not yet known. Energy balance analysis in lactating cows shortly after post-calving revealed that the majority of the cows exhibited transiently negative energy balance levels, whereas the remaining cows exhibited either consistently negative or consistently positive energy levels. Metabolic status was associated with EV-coupled miRNA composition in the follicular fluid. Cows experiencing NEB showed reduced expression of a large number of miRNAs while cows with positive energy balances primarily exhibited elevated expression of EV-coupled miRNAs. The miRNAs that were suppressed under NEB were found to be involved in various metabolic pathways. This is the first study to reveal the presence of an association between EV-coupled miRNA in follicular fluid and metabolic stress in dairy cows. The involvement of differentially expressed miRNAs in various pathways associated with follicular growth and oocyte maturation suggest the potential involvement of specific follicular miRNAs in oocyte developmental competence, which may partially explain reduced fertility in cows due to post-calving metabolic stress.

## Introduction

Selection for milk yield in dairy cows in the last 70 years has resulted in a significant increase in milk yield and concomitant reduction in fertility. In modern dairy cows, heifers have calving rates of ~55–60% that reduce to ~35–40% in lactating cows^1^. One of the main causes of reduced fertility rates is postpartum negative energy balance (NEB) during early lactation, a condition in which a cow’s energy demand for maintenance and lactation is higher than its dietary energy intake. Negative energy balance have been documented to have the strongest association with declining fertility^2–5^. During NEB, cows have a shortage of circulating glucose and their body starts to metabolize fat reserves through lipogenesis and/ketogenesis. This leads to an increase of undesired substances in the blood such as non-esterified fatty acids (NEFA) and beta-hydroxybutyrate, which are known to have a negative effect on folliculogenesis^5,6^. This disturbance in blood metabolite levels is then directly reflected onto the follicular fluid microenvironment^7^. As a consequence, in the first few months of the post-calving period, ovarian follicle development occurs in a compromised endogenous metabolic microenvironment^6^ and results in significantly less viable preantral follicles^8^.

Follicular fluid (FF) provides a nurturing microenvironment for the development of oocytes by allowing them access to various nutrients and hormones that are produced from surrounding somatic cells. Follicular fluid is a product of both blood plasma constituents that cross the blood follicular barrier and secretions from granulosa and theca cells^9^. Studies utilizing gas chromatography mass spectrometry to assess FF metabolite levels have reported higher concentrations of NEFA and b-hydroxybutyrate and lower concentrations of glucose, insulin, and Insulin-like growth factor in lactating cows compared to non-lactating cows and heifers^10^. In addition, supplementation of NEFA during *in vitro* oocyte maturation had a negative effect on maturation, fertilization, cleavage, blastocyst rates, and number of late apoptotic cumulus cells^11^. Moreover, environmental factors like heat stress are also known to aggravate the consequences of NEB in high yielding dairy cows by altering biochemical concentrations in the follicular fluid of dominant follicles with profound effects on oocyte and granulosa cell quality^12^. Since oocyte quality is known to determine the number of transferable blastocysts, identification of the best quality oocytes prior to IVF has been the main focus in the field of assisted reproductive technology^9,13^. As such, biochemical reactions like brilliant cresyl blue staining^13^ and morphological parameters are utilized to select oocytes invasively. Therefore, molecules such as DNA, mRNA, miRNAs, lipids, and proteins that are released into follicular fluid from surrounding follicular cells via extracellular vesicles during the cell-to-cell communication could serve as non-invasive molecular markers for oocyte competence.

Extracellular vesicles (EVs) are evolutionarily conserved nano-sized cargo-carrying molecules released from both prokaryotic and eukaryotic cells to deliver signals to target cells^14,15^. Extracellular vesicles have been detected in various biological fluids including nasal mucosal fluid^16^, cerebrospinal fluid^17^, breast milk^18^, saliva^19^, umbilical cord blood^20^, urine^21,22^, amniotic fluid^22^, bovine follicular fluid^23,24^ and semen^25^. Extracellular vesicle is a general term encompassing several different vesicle types, including exosomes, microvesicles, apoptotic vesicles, and, in pathological situations, necrotic debris. They can be released by cells constitutively or in response to specific stimuli or cell stressors^26^. Most studies have primarily concentrated on the content of small nano-sized vesicles: exosomes and ectosomes. Exosomes are formed from internalized endocytic vesicles and are constitutively secreted from the cell^26^ whereas ectosomes are ubiquitous vesicles assembled at and released from the plasma membrane^27^. Extracellular vesicles play a vital role in cell-to-cell communication and carry a huge number of proteins, lipids, mRNA, and microRNAs (miRNAs) that can be delivered to and function in other cells^15^. Thousands of exosome-mediated molecules have been documented, including about 9,769 Proteins, 3,408 mRNAs, 2,838 miRNAs, and 1,116 lipids^28^. In the early stages of the human reproductive process, the ovarian follicle, seminal fluid, endometrium, embryo, and trophoblast cells are all possible sources of EVs that have the potential to locally modulate maternal immune function^26^. Extracellular vesicle-mediated miRNAs have been detected in the follicular fluid of bovine^23^, human^29^, and other species. We and others previously determined that exosomal miRNAs in bovine and human follicular fluid are associated with developmental potential of oocytes^23,29^. Due to extensive cell-to-cell communication in the follicular environment, we postulate that metabolic stress can greatly affect EV-mediated release of molecules and subsequently follicular growth and oocyte maturation. Therefore, the aim of this study was to investigate the association between the expression of EV-coupled miRNAs in bovine follicular fluid and postpartum metabolic stress in Holstein-Friesian cows by comparing metabolically stressed vs non-stressed cows. Furthermore, we aimed to investigate the effect of lactation physiology on the expression of EV-coupled miRNAs in follicular fluid by comparing metabolically non-stressed lactating cows versus heifers.

## Results

### Quantification of blood serum metabolites

**Figure 1:**
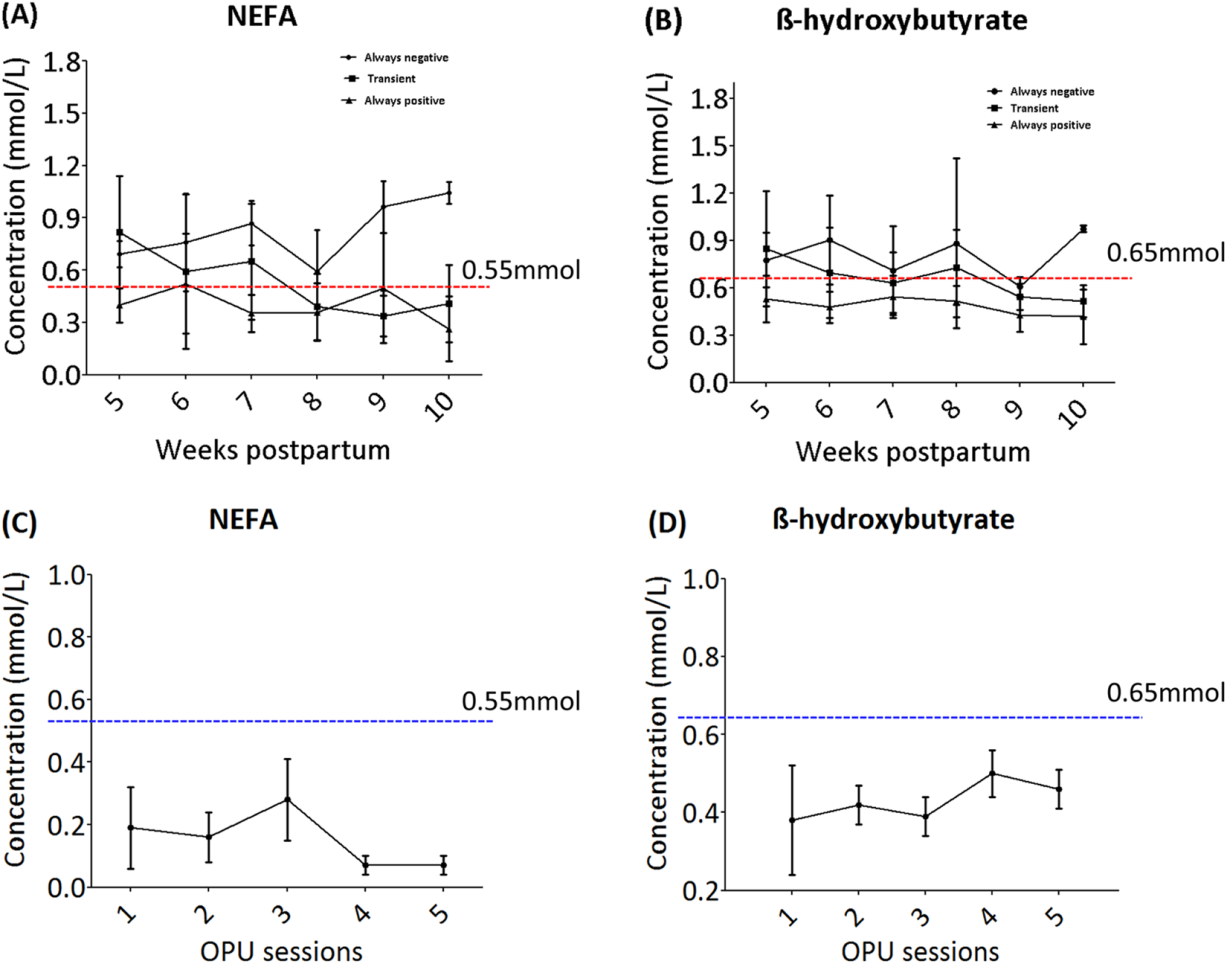
Blood serum metabolite profiles of postpartum cows and heifers. (**A**) The average weekly based concentration of NEFA for different metabolic status of population of postpartum cows early post-calving (weeks 5–10). **(B)** The average weekly based concentration of ß-hydroxybutyrate for different metabolic status of postpartum cows (weeks 5–10). **(C)** The average concentration of NEFAs per OPU session for control heifers. (**D)** The average concentration of ß-hydroxybutyrate per OPU session for heifers. The dashed lines in all graphs indicate the threshold level of metabolites to categorize animals as having negative or positive energy balance. Always negative cows: cows that have high concentration of NEFA and ß-hydroxybutyrate throughout the experimental period weeks 5–10. Transient cows: cows that have high concentration of NEFA and ß-hydroxybutyrate at week 5–6 and reduce to very low level starting from weeks 7–8 until week 10. Always positive cows: cows that have low concentration of NEFA and ß-hydroxybutyrate throughout the experimental period week 5–10.

In the present study, the energy status of experimental cows and heifers was assessed using blood metabolite analysis. Non-esterified fatty acids (NEFA) and Beta-hydroxybutyrate (β-OHB) were profiled at different time points post-calving. As shown in Fig. 1A, average NEFA concentration for experimental cows decreased from 0.73 ± 0.0.31 mmol/L at week 5 to 0.47 ± 0.032 mmol/L at week 10. Similarly, the concentration of ß-OHB decreased from 0.78 ± 0.33 mmol/L at week 5 to 0.57 ± 0.21 mmol/L at week 10 as shown in Fig. 1B. The average NEFA and ß-OHB concentrations in heifers were ≤0.2 mmol/L and ≤0.5 mmol/L, respectively, as shown in Fig. 1C,D. Moreover, individual variations were observed among the experimental cows and 66% (20/30) transiently exhibited high concentrations of NEFA and ß-OHB, whereas 16% of the cows consistently exhibited high concentration of both metabolites at all time-points of the analysis. Interestingly, 16% of the cows exhibited lower concentrations of NEFA and ß-OHB through-out the early lactation period (weeks 5–10). Cows were considered to be in negative energy balance when NEFA and ß-OHB concentrations in serum were >0.55 mmol/L and >0.65 mmol/L, respectively, as applied previously^30^^,^^31^.

**Figure 2:**
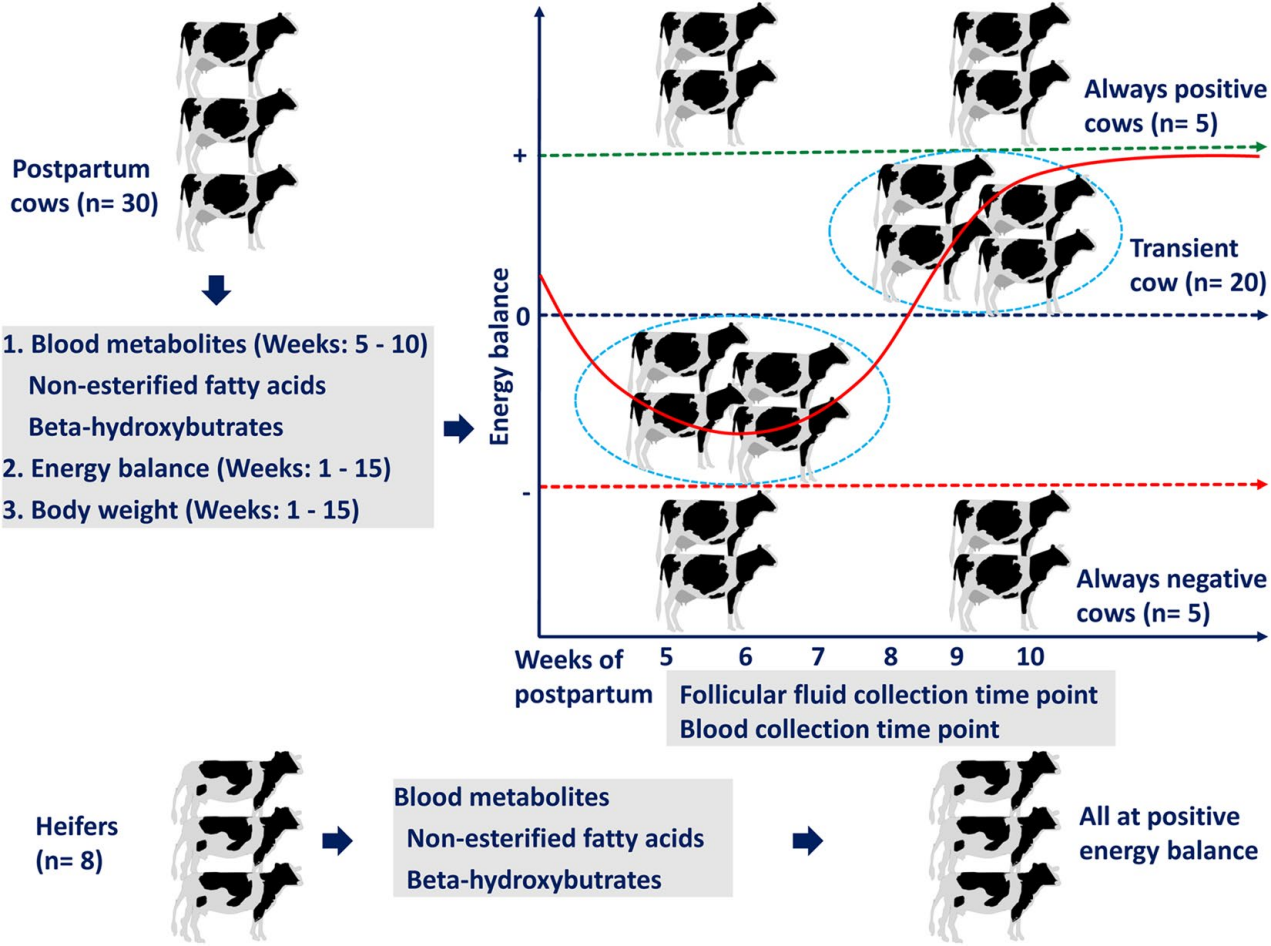
Summary of different categories of cows based on energy status as determined from blood serum metabolites, energy balance, and body weight curve of individual animals. Blood serum metabolites were measured weekly weeks 5 to 10. Individual cow body weight was measured daily (Supplementary Fig. S1). The overall energy balance of individual cows was measured based on their dry matter intake on a daily basis (Supplementary Fig. S2).

In addition to metabolic profiles, cow body weight measurements were performed during the experimental period and body weight curves were generated as indicated in Supplementary Fig. S1. Moreover, measurement of energy status of individual cows was performed based on dry matter intake and energy expenditure for milk yield and maintenance. Based on their feed intake and milk yield the energy status of individual cows was calculated as follows. Energy balance (EB) (MJ NEL/d) = Energy intake (EI) − Energy consumption (EC). Individual cow energy balances are available as Supplementary Fig. S2. Based on the combination of metabolic profiles, body weight curves, and energy balances between weeks 5 and 10 post-calving, all experimental cows were categorized into three groups: 1) Always negative cows (ANCs): cows that showed negative energy balance at all times between weeks 5 and 10; 2) Always positive cows (APCs): cows that showed positive energy balance at all times between weeks 5 and 10; 3) Transient cows (TCs): cows which showed negative energy balances at weeks 5–6 and recovered at weeks 9–10 by showing positive energy balance. While 20 out of 30 (66%) of the postpartum cows were ‘transient cows’ (TCs), each ANC and APCs group represents 17% (five out of 30). The distribution of the experimental animals based on the energy status is illustrated in Fig. 2.

### Characterization of extracellular vesicles and isolated RNAs

**Figure 3:**
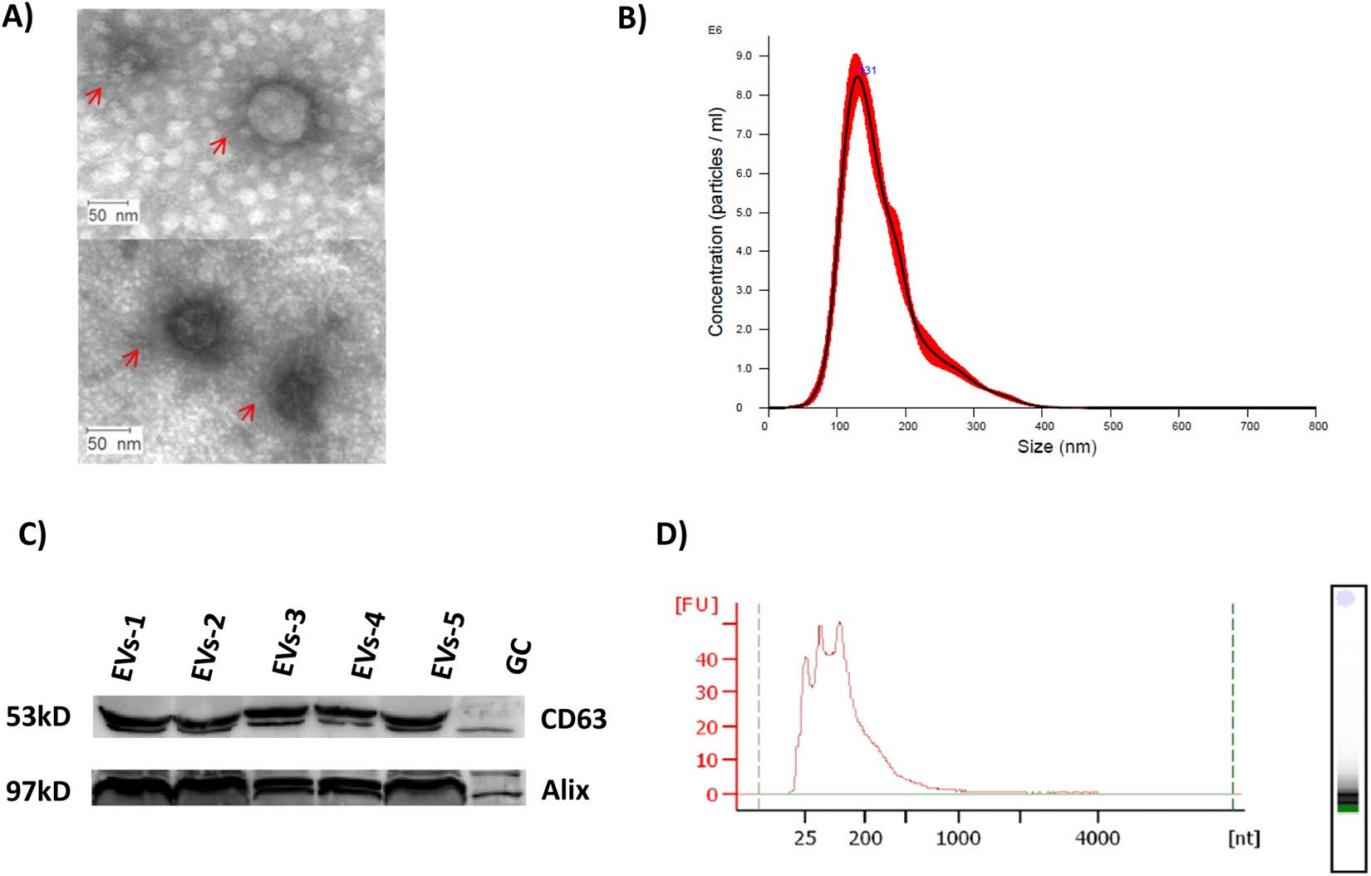
Morphological and molecular characterization of EVs recovered from follicular fluid. (**A**) Electron microscope image of EVs. The red arrow shows the EVs morphology. (**B**) A representative nanoparticle tracking analysis of EVs. (**C**) Detection EV protein markers. The numbers represent different sources EVs 1: EVs of TCs (weeks 5–6; n = 11), 2: EVs of TCs (weeks 9–10; n = 7), 3: EVs of ANCs (n = 2), 4: EVs of APCs (n = 2), 5: EVs of heifers (n = 8) and GS: Granulosa cells and the full image indicated in Supplementary Fig. S3. (**D**) A representative electrophorogram image of EVs RNA quality analysis using Agilent bioanalyser.

Following isolation of EVs by ultra-centrifugation, morphological characterization of EVs was done using transmission electron microscope as shown in Fig. 3A. Furthermore, the concentration and the size distribution of extracellular vesicles isolated from follicular fluid of cows with different statuses were quantified using a nanoparticle tracking system. The size of follicular fluid EVs was found to be within the range of 124.8 ± 2.4 to 147.1 ± 5.6 nm in diameter as shown in Fig. 3B. The concentration of particles recovered from follicular fluid was between 4.43e + 008 ± 1.13e + 008 and 2.39e + 009 ± 5.01e + 007 particles/ml. The specificity of EVs isolated from follicular fluid from each experimental group was evidenced by detection of EV protein markers, namely CD63 and Alix, using protein specific antibodies as indicated in Fig. 3C. Extracellular vesicle’s RNA quality assessment showed the absence of 18 s and 28 s bands, which indicate the absence of cellular RNA contamination (Fig. 3D). The purity of total RNA isolated from EVs was determined using Nanodrop and a 260/280 ratio of 1.25–1.55 was obtained as shown in Supplementary Table S1. Moreover, the measurements of 260/230 ratio were found to be below 0.5 for all samples.

### Sequence quality and mapping of RNAseq data from different small RNA samples

Next generation sequencing was performed using the Illumina-based NextSeq. 500 instrument with an average number of 12 Million reads per sample for which 75 nucleotide single-end reads were retained. Quality control of the sequenced data was performed before and after mapping for all biological replicates. Sequenced raw data for all replicates had a Q-score above 30 (<1 incorrect base call 1 every 1000 bases). Following this, mapping of the sequencing data was performed against the reference sequence. Sequencing of EV-coupled small RNAs generated an average 6.7 million reads per sample and the average genome mapping rate was 37.7%. The raw sequencing reads and the processed data have been deposited in NCBI’s Gene Expression Omnibus with GEO accession number GSE129367.

### Global detection of EVs mediated miRNAs and small RNAs in follicular fluid of different metabolic status cows

**Figure 4:**
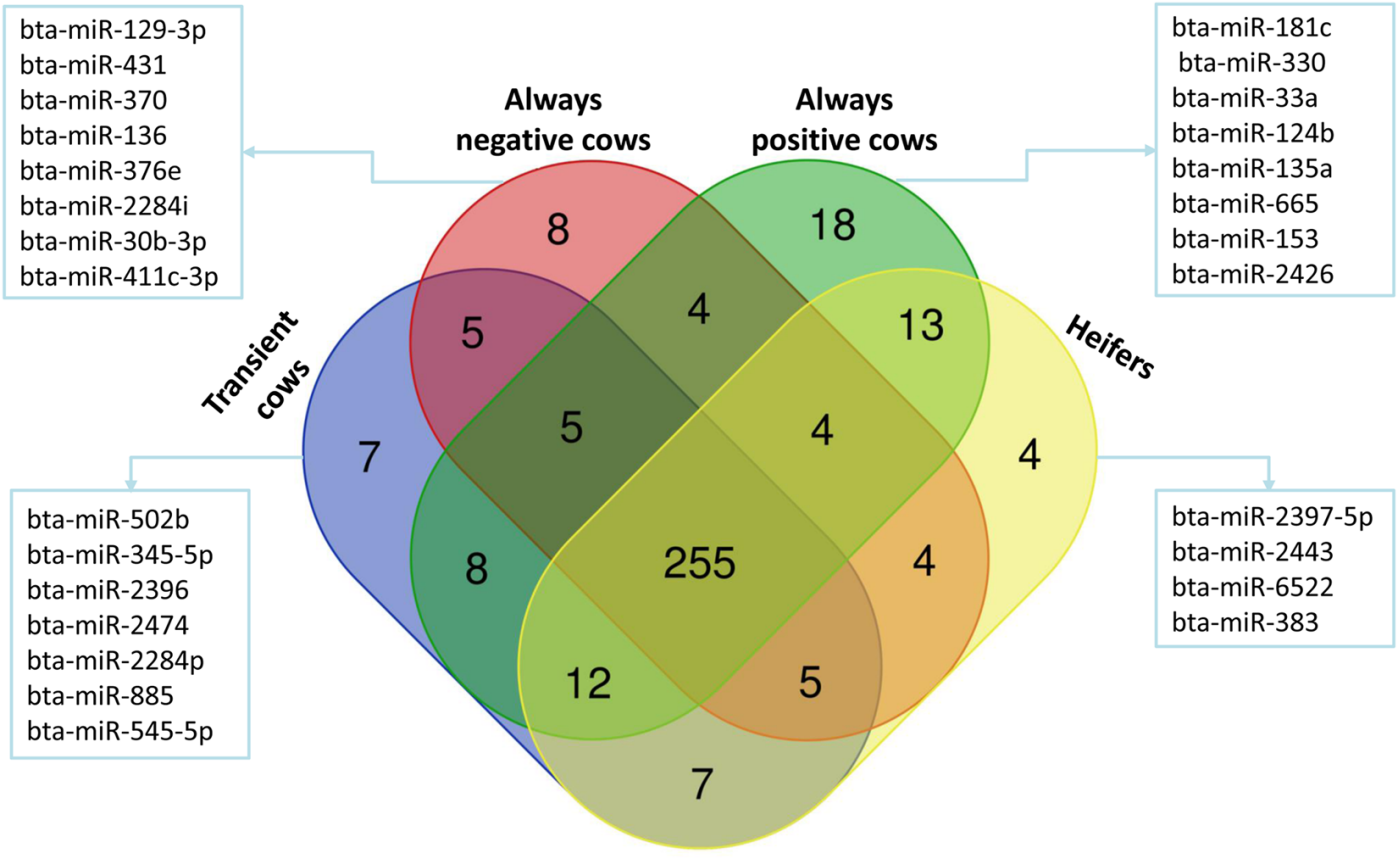
Venn diagram showing unique and shared detection of miRNAs in TCs, ANCs, APCs and heifers. For a miRNA to be considered as detected, we use a read count of at least 1 and above throughout all biological replicates.

Sequencing results showed that a total of 356 known miRNAs were detected across all analyzed samples, 255 of which were commonly detected in all experimental groups as shown in Fig. 4. However, some miRNAs were uniquely detected in specific experimental groups and the highest numbers of unique miRNAs were detected in APC samples. APC samples had 18 unique miRNAs, including bta-miR-2400, bta-miR-181c, bta-miR-2284k, bta-miR-2285n, bta-miR-2285j, bta-miR-2284ac, bta-miR-2285v, bta-miR-330, bta-miR-2436-5p, bta-miR-2284v, bta-miR-33a, bta-miR-124b, bta-miR-2382-3p, bta-miR-135a, bta-miR-2285ab, bta-miR-665, bta-miR-153 and bta-miR-2426. Moreover, eight, seven and four unique miRNAs were detected in ANCs, TCs, and heifers, respectively.

### Hierarchical clustering of EV-coupled miRNA in cows of different metabolic status

**Figure 5:**
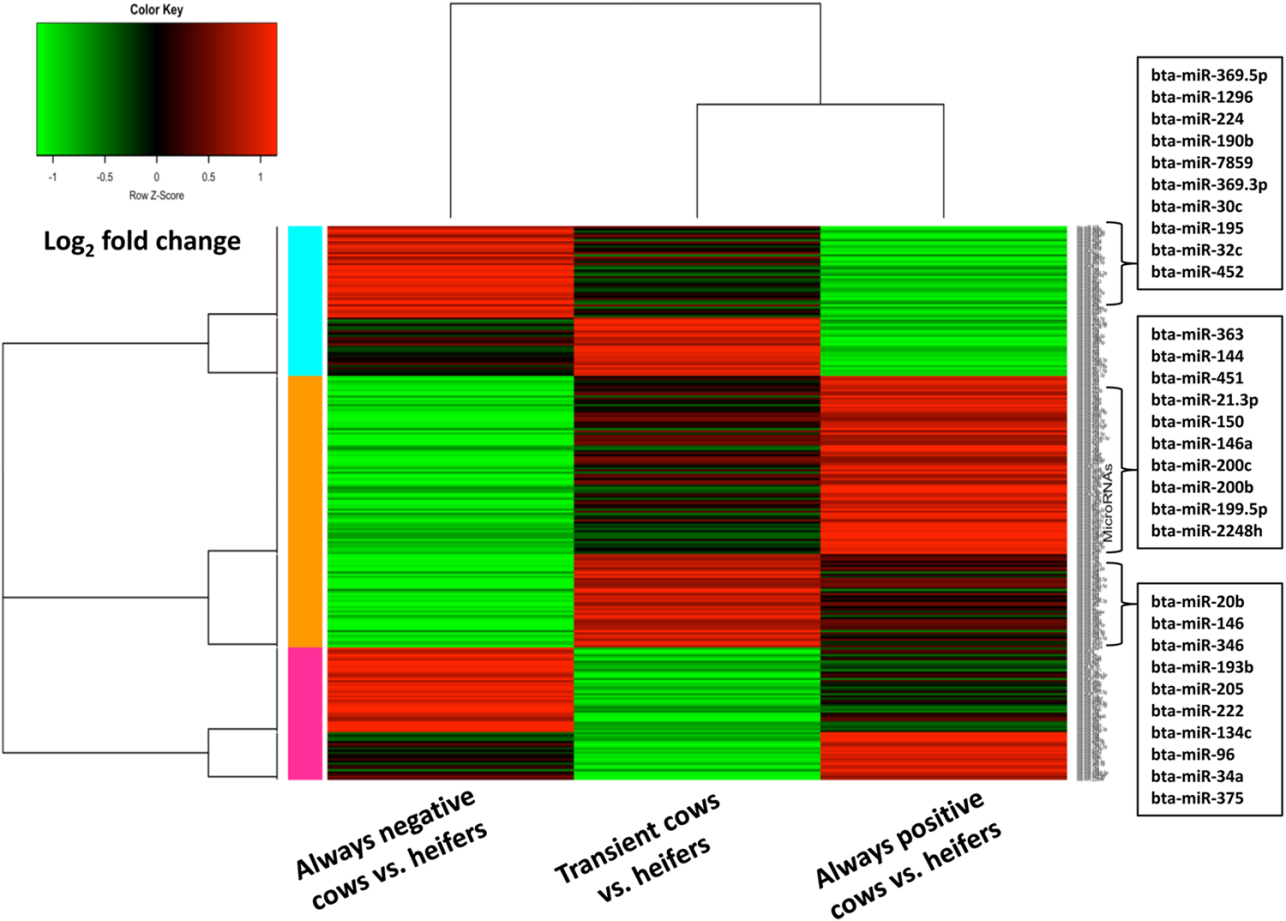
Heat map of differentially expressed EV miRNAs in follicular fluid of cows of different metabolic status at weeks 5 and 6 compared to heifers. The log_2_ fold change of each miRNA in different pairwise comparisons were used to create the heat map using R-studio package.

Differential expression analysis of miRNAs recovered from follicular fluid of cows in different metabolic status revealed that cows with different metabolic statuses exhibited different miRNA expression profiles. Pairwise comparisons between APCs, ANCs, and TCs and control heifers determined that the differentially expressed (DE) miRNA profiles of TCs and APCs were relatively similar whereas ANCs exhibited a more distinct DE miRNA profile (Fig. 5). Namely, most of the DE miRNAs were downregulated in ANCs while a large proportion of the TCs and APCs DE miRNAs exhibited induced expression compared to control heifers.

**Figure 6:**
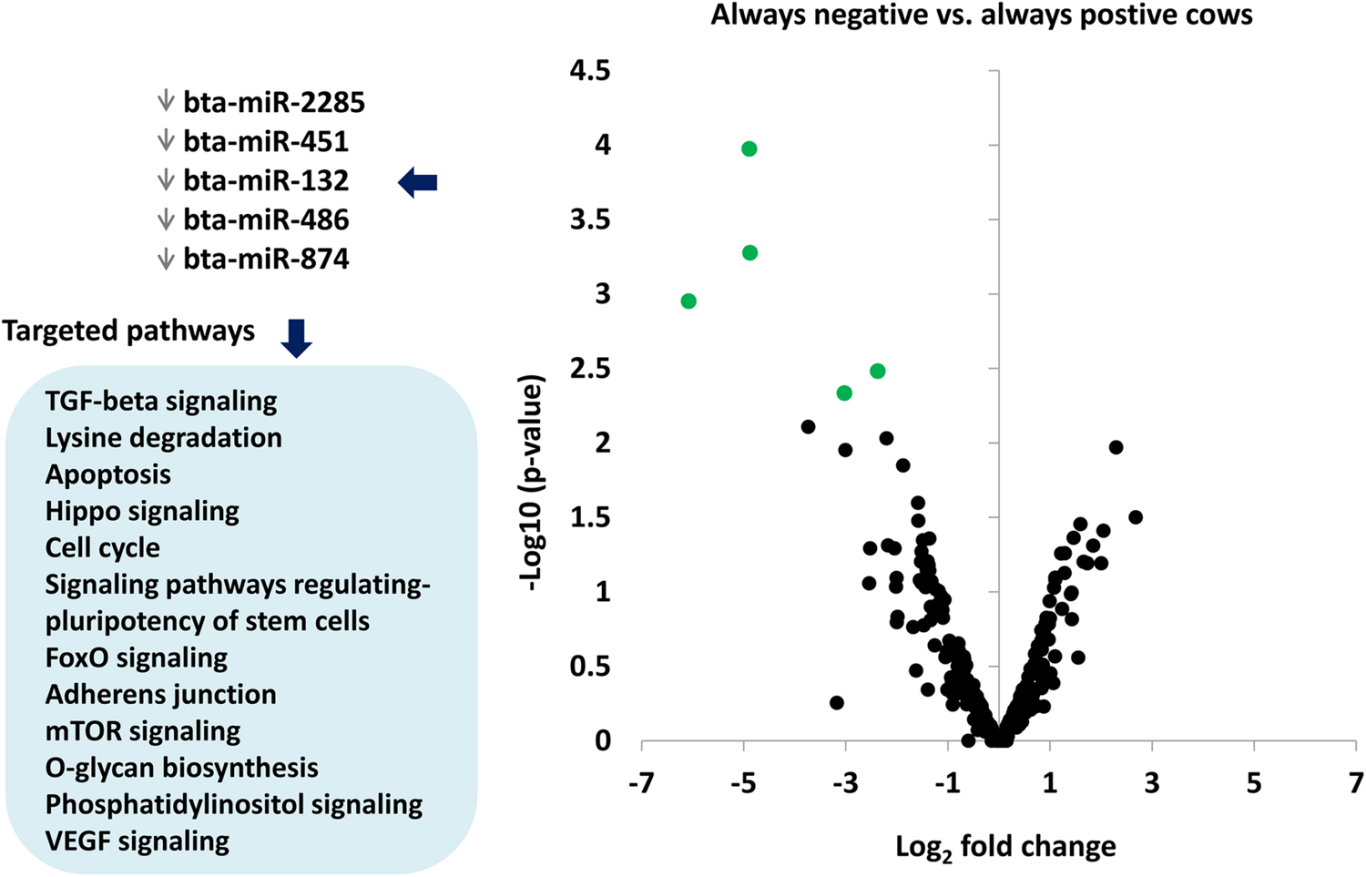
Volcano plot of differentially expressed EV-coupled miRNAs in follicular fluid of ANCs compared to APCs and the enriched pathways targeted by downregulated miRNAs. Downward arrows indicate downregulation in expression in ANCs compared to APCs (Details of raw read count and FDRs are presented in Supplementary Table S2).

In order to identify miRNAs differentially abundant in EVs derived from cows which consistently exhibited negative or positive energy status, the miRNA expression profiles of ANCs and APCs were compared in a pairwise fashion. Interestingly, all five differentially expressed miRNAs were downregulated in cows that persistently remained in metabolic stress condition compared to cows always in positive energy status (Fig. 6). Target prediction of those downregulated miRNAs revealed their involvement in various pathways associated with ovarian function including apoptosis, hippo signaling, TGF-beta signaling, lysine degradation, cell cycle, FoxO signaling, mTOR signaling and others (Fig. 6).

Furthermore, pairwise comparison of the miRNA expression profiles of ANCs to metabolically unstressed heifers showed differential expression of 37 miRNAs, of which 25 miRNAs were downregulated and only 12 miRNAs were upregulated (Table 1). Moreover, target prediction and subsequent pathway analysis for downregulated miRNAs revealed pathways involved in different metabolic processes and oocyte follicular growth, including insulin signaling, estrogen signaling, MAPK signaling, vitamin B6 metabolism, fatty acid biosynthesis, fatty acid metabolism, fatty acid elongation, progesterone-mediated oocyte maturation and others as indicated in the Supplementary Fig. S4. This may indicate that impaired folliculogenesis due to metabolic stress in lactating cows could be associated with disturbed miRNA expression in follicular cells, which is reflected in the level of EV-coupled miRNAs released into the follicular fluid. Moreover, miRNA gene network analysis revealed the involvement of candidate miRNAs and their target genes in the interaction network as indicated in Supplementary Fig. S5.

**Table 1:** List of differentially expressed EV-coupled miRNAs in follicular fluid of ANCs versus heifers.

MiRNA name	Normalized TMM read counts		Log_2_ Fold change	p value	MiRNA name	Normalized TMM read counts		Log_2_ Fold change	p value
	Always negative	Heifers				Always negative	Heifers		
bta-miR-20b	0	21.23	−5.57	0.0037	bta-miR-708	30.51	93.28	−1.62	0.0226
bta-miR-363	0	18.68	−5.34	0.0059	bta-miR-200c	924.02	2805.82	−1.61	0.0011
bta-miR-132	93.73	2719.73	−4.85	0.0001	bta-miR-200b	1370.16	4067.04	−1.57	0.0016
bta-miR-184	174.65	2454.35	−3.84	0.0005	bta-miR-432	279.09	738.26	−1.43	0.0202
bta-miR-451	20.05	228.61	−3.77	0.0001	bta-miR-182	240.47	598.4	−1.31	0.0095
bta-miR-223	125.45	875.5	−2.84	0.0000	bta-miR-191	7774.03	17291.16	−1.15	0.0181
bta-miR-18a	14.1	76.19	−2.66	0.0035	bta-miR-125a	11060.62	5530.14	1.00	0.0252
bta-miR-122	1924.24	12098.39	−2.66	0.0036	bta-miR-26a	14872.37	7174.03	1.05	0.0204
bta-miR-147	10.09	56.61	−2.65	0.0069	bta-miR-423-5p	62976.07	30282.96	1.06	0.0194
bta-miR-21-3p	6.08	37.25	−2.59	0.0291	bta-miR-450b	1624.61	776.18	1.07	0.0142
bta-miR-150	181.84	914.97	−2.37	0.0002	bta-miR-328	455.07	215.61	1.08	0.0209
bta-miR-146a	86.91	376.36	−2.18	0.0014	bta-miR-92a	60915.34	27486.24	1.15	0.0145
bta-miR-138	6.2	38.39	−2.17	0.0121	bta-miR-92b	6096.23	2558.5	1.25	0.0254
bta-miR-193b	4.13	26.48	−2.15	0.0242	bta-miR-6520	117.92	46.65	1.30	0.0288
bta-miR-155	325.07	1375.11	−2.09	0.0006	bta-miR-542-5p	388.66	151.21	1.35	0.0060
bta-miR-142-5p	583.69	2285.45	−1.98	0.0016	bta-miR-1306	393.91	139.79	1.51	0.0056
bta-miR-205	1000.1	3541.22	−1.83	0.0079	bta-miR-424-5p	2015.21	654.03	1.62	0.0006
bta-miR-183	176.76	619.32	−1.82	0.0049	bta-miR-6518	71.36	21.97	1.70	0.0140
bta-miR-206	30.76	109.02	−1.70	0.0151					

Extended list of miRNAs with the corresponding raw read count and FDRs are presented in Supplementary Table S3.

### EV-coupled miRNAs as predictors of transition from negative energy to positive energy status during early lactation

The follicular fluid EV-coupled miRNA profiles of ANCs and TCs at weeks 5 & 6 was compared to determine candidate miRNAs that may be indicators for the transition to positive energy balance in the later stages of lactation (weeks 9 and 10). In this comparison, miRNAs (i.e., bta-miR-34b, bta-miR-34c, bta-miR-449a, and bta-miR-132) were downregulated in ANCs compared to TCs, which have recovered from their metabolic stress at weeks 9 & 10. Further, comparison of DE miRNAs between EVs recovered from follicular fluid collected from transient cows at weeks 5–6 and weeks 9–10 revealed that bta-miR-34b, bta-miR-34c, and bta-miR-449a were upregulated at the time of recovery at week 9–10, whereas two miRNAs, bta-miR-451 and bta-miR-592, were downregulated at weeks 9–10.

### Identification of EV-coupled miRNAs induced or suppressed by lactation physiology

In order to determine the effect of lactation on EV-coupled miRNA release in follicular fluid, the miRNA expression profiles of APCs versus heifers were compared. Both groups are metabolically unstressed and any differences in miRNA expression would likely relate their different lactation physiologies. Our results revealed that out of 38 differentially expressed miRNAs, 31 miRNAs were upregulated and only seven miRNAs were downregulated in APCs or lactating cows compared to heifers (Table 2). Target prediction of lactation-induced miRNAs revealed their involvement in various biological pathways, including gap junction, PI3K-Akt signaling, steroid biosynthesis, fatty acid degradation, ErbB signaling, AMPK signaling, sphingolipid signaling, central carbon metabolism, insulin signaling, phosphatidylinositol signaling system, prolactin signaling, MAPK signaling, ras signaling, estrogen signaling, fatty acid metabolism, fatty acid elongation, mTOR signaling, p53 signaling, focal adhesion, Wnt signaling and others as indicated in the Supplementary Fig. S6.

**Table 2:** List of differentially expressed EV-coupled miRNAs in follicular fluid of APCs compared to heifers.

MiRNA name	Normalized TMM read counts		Log_2_ Fold change	p value	MiRNA name	Normalized TMM read counts		Log_2_ Fold change	p value
	Always positive	Heifers				Always positive	Heifers		
bta-miR-135a	17.13	0.86	3.72	0.0039	bta-miR-6520	145.68	40.8	1.81	0.0029
bta-miR-2285z	34.14	2.38	3.45	0.0001	bta-miR-6518	66.63	19.22	1.78	0.0111
bta-miR-502a	145.84	16.32	3.09	0.0000	bta-miR-2366	95.69	28.92	1.68	0.0084
bta-miR-449a	170.79	21.4	2.95	0.0240	bta-miR-423-5p	82036.05	26419.48	1.63	0.0011
bta-miR-874	230.75	30.7	2.94	0.0000	bta-miR-335	1834.38	614.55	1.58	0.0292
bta-miR-1291	17.13	2.31	2.92	0.0124	bta-miR-652	310.1	105.7	1.55	0.0035
bta-miR-1246	33974.57	4846.76	2.81	0.0000	bta-miR-671	376.02	129.93	1.53	0.0029
bta-miR-320b	22.21	3.76	2.75	0.0085	bta-miR-2284 ×	13219.16	4627.82	1.51	0.0028
bta-miR-130a	23627.76	4333.53	2.45	0.0007	bta-miR-26a	17422.87	6256.15	1.48	0.0024
bta-miR-331-5p	32.61	5.64	2.44	0.0031	bta-miR-6517	189.85	70.22	1.46	0.0113
bta-miR-23b-3p	38460.93	7357.16	2.39	0.0000	bta-miR-92b	5913.8	2234.63	1.40	0.0251
bta-miR-107	45710.3	9316.24	2.29	0.0001	bta-miR-296-3p	4994.23	2302.28	1.12	0.0261
bta-miR-362-3p	32.36	5.61	2.28	0.0045	bta-miR-204	44.67	126.49	−1.50	0.0163
bta-miR-873	9540.4	2009.85	2.25	0.0077	bta-miR-194	575.71	1742.56	−1.60	0.0120
bta-miR-320a	148650.4	31784.41	2.23	0.0001	bta-miR-205	895.08	3078.37	−1.78	0.0022
bta-miR-103	44908.49	10770.95	2.06	0.0003	bta-miR-196b	22.33	130.08	−2.53	0.0032
bta-miR-23a	47898.5	11874.35	2.01	0.0001	bta-miR-196a	17.38	152.79	−3.12	0.0003
bta-miR-22-3p	9333.16	2434.79	1.94	0.0003	bta-miR-184	140.03	2137.16	−3.93	0.0000
bta-miR-1839	13301.78	3474.36	1.94	0.0009	bta-miR-365-5p	0	16.04	−5.34	0.0057

Extended list of miRNAs with the corresponding raw read count and FDRs are presented in Supplementary Table S4.

After performing pairwise comparisons between ANCs and APCs and control heifers, we then compared the DE miRNA profiles of ANCs and APCs to identify commonly differentially expressed miRNAs. These common DE miRNAs may represent circulatory miRNAs that may have been induced or suppressed due to lactation-induced metabolic stress. Out of the seven common miRNAs, miR-205 and miR-184 were suppressed and miR-92b, miR-6518, miR-6520, miR-26a, and miR-423-5p were upregulated.

## Discussion

In this *in vivo* study, the association between postpartum metabolic physiology and EV-coupled miRNAs expression in the follicular fluid of the antrum follicle has been investigated in Holstein Fresian cows and heifers. Previous studies have shown that increased NEFA and ^β^-hydroxybutyrate concentration in the blood stream of metabolically stressed cows resulted in disturbed follicular microenvironments^7^. This phenomenon of negative energy balance has been evidenced to have a huge negative impact on follicular cell development, quality of oocytes, and embryo survival. However, the molecular mechanisms associated with metabolic stress in dairy cows are poorly understood. In the present study, we have demonstrated the association between metabolic status and EV-coupled miRNA profiles in the follicular fluid of postpartum cows. These changes could partly signify EV-mediated cell-to cell communication between the oocyte and the surrounding cell in antrum follicles with respect to the molecular messages carried by those vesicles. Moreover, several potential candidate miRNAs have been identified, which are not only indicators of negative energy status, but are potential biomarkers for the transition to positive energy status in cows, as shown in TCs. In order to observe the association between postpartum negative energy balance and EV-coupled miRNA signatures in follicular fluid, we analyzed individual cows’ metabolic statuses to get a clear demarcation between the experimental groups with respect to the energy status. Overall, we have detected the presence of high blood serum NEFA levels (0.73 ± 0.0.31 mmol/L and high ^β^-hydroxybutyrate (0.78 ± 0.33 mmol/L), along with body weight loss and lower overall energy (MJ) during the early stage of lactation for the majority of the cows. According to previous studies, lactating cows with an average concentration of NEFA >0.55 mmol/L^30^ and β-hydroxybutyrate >0.65 mmol/L^31^ in the blood serum are indicators of negative energy balance. While only one fourth of the cows showed a positive energy balance in early period of lactation, increased blood serum NEFA and ^β^-hydroxybutyrate was evident as sign of clinical metabolic status due to lactation during early postpartum for most of the cows. The majority of cows (66%) showed a negative energy balance during their 5–6 weeks postpartum, but they recovered from their metabolic stress in the later stages of the analysis. However, some of the cows (16%) continued to exhibit negative energy balances until the later stages of the analysis. Interestingly, a similar proportion of cows showed no sign of metabolic stress throughout the analysis period. These results revealed individual differences between cows in terms of dealing with metabolic stress during early lactation. Molecular genetic analysis of such divergent groups of cows will allow us to understand the molecular mechanism associated with metabolic stress due to lactation and paves the way for identification of metabolic markers and development of future therapeutic strategies to tackle negative effects from metabolic stress in dairy cow reproductive efficiency.

The EV isolation methodology used in the present study was evaluated based on the size distribution and morphology of the isolated EVs. Moreover, nanoparticle tracking analysis revealed the majority of the EVs were as big as 124.8–147.1 nm in diameter, indicating absence of contamination of large vesicles during EV isolation. Following morphological characterization, CD63 and Alix protein markers, which are known to be enriched in exosomes from virtually any cell type, were used to determine the specificity of our EV isolation procedure^32^. The 260/280 and 260/230 ratio of total RNA isolated from EVs were found to deviate from cellular RNAs readings, which can be attributed to the source of biological samples as it has been evidenced previously^33^. In the same study RNA isolated from urine EVs were found to have significantly different characteristics compared to their cellular counterparts. We have analyzed EV-coupled small RNAs including miRNA with a read length of 75nt. The read length distribution for miRNAs was observed to peak around 18–23nt. Our result revealed that a huge number of known miRNAs (365) and putative miRNAs (158) were detected in EVs recovered from follicular fluid from follicles >8 mm. The detected miRNAs accounted for 48% of the 762 known bovine miRNAs available in mirbase_20−. Considering that about 70% of the total detected miRNAs were found in all experimental groups it suggests they have housekeeping roles in cell-to-cell communication as mediated by EVs in the follicular environment. The housekeeping role of several miRNAs in ovarian granulosa cells from dominant and subordinate follicle has been reported previously^34^. Moreover, sequence analysis revealed the presence of unique EVs-coupled miRNAs in the follicular fluid of cows with divergent metabolic status. As shown in Fig. 4, cows that had negative energy status throughout the experimental period showed exclusive expression of eight miRNAs, five out of which (bta-miR-431, bta-miR-370, bta-miR-136, bta-miR-376e and bta-miR-411c-3p) were found on chromosome 21. A comparable number of miRNAs have been uniquely detected in transiently negative energy cows and heifers. However, the numbers of uniquely expressed miRNAs were highest in cows that consistently exhibited positive energy balances throughout the experimental period. Four of these miRNAs (i.e., miR-2436-5p, bta-miR-33a, bta-miR-135a and bta-miR-2426) were found on chromosome 5, which suggests their importance in reproduction. A previous study showed that deletion between 25 and 70 Mb on chromosome 5 was associated with decreased reproductive efficiency in female cattle^35^. Two of the uniquely detected miRNAs, bta-mir-135a and bta-mir-2426, are found in the same 25 and 70 Mb region. Moreover, the well-studied miR-181 gene family, which was uniquely detected in APCs, is a cellular metabolic rheostat essential for NKT cell development and regulates homeostasis in NKT cell ontogenesis and lymphocyte development in mice^36^. Also, miR-181a regulates lipid metabolism, as miR-181a transgenic mice have lower body weights and less lipid accumulation compared to wild-type^37^. Therefore, the detection of the miR-181 family exclusively in the cows with consistently positive energy balance may be associated with metabolic homeostasis.

Hierarchal clustering of differentially expressed EV-coupled miRNAs between cows with divergent metabolic statuses revealed a clear association between metabolic stress and the release of EV-coupled miRNAs. As shown in Fig. 4, TCs and APCs have relatively similar expression patterns, while ANCs exhibited reduced expression of a significant number of EV-coupled miRNAs. Previous studies have shown the association between glucose level and expression of miRNAs in human mesenchymal stem cells^38^, in which higher glucose levels suppressed the expression of candidate miRNAs. The reduced release of EV-coupled miRNAs in the follicular fluid of metabolically stressed cows in the present study could be associated with reduced glucose concentration in those animals. This has been also evidenced in the direct comparison of always negative versus always positive cows for which a large set of known and putative miRNAs were found to be globally suppressed in the always negative energy cows. This suggests metabolites in the follicular fluid do have a direct regulatory role in the expression of miRNAs in follicular cells and their subsequent release into the extracellular environment. Even though the mechanism behind the response of the genome to metabolic changes is not fully understood, specialized transcription factors have been documented to be activated in response to metabolic changes and result in gene expression alterations^39^. Hormonal regulation of miRNAs is a well-documented fact in various mammalian species. The expression of miRNAs including miRNA-183, miRNA-132 and miR-122, which are among candidates in the present study, have been reported to be altered after treatment 17ß-estradiol in Rat granulosa cells^40^. Interestingly, early postpartum dairy cows with metabolic stress have been documented to have very law oestradiol concentrations^41^. Therefore, the major suppression of EVs-coupled miRNA in the current study could be associated to the law level of oestradiol and suggests metabolites in the follicular fluid do have a direct regulatory role in the expression of miRNAs in follicular cells and their subsequent release into the extracellular environment. Similarly, supplementation of elevated NEFA concentrations during oocyte maturation *in vitro* found to alter the expression level of DNMT3A, IGF2R and SLC2A1 genes in day 7 blastocysts^42^.

In the current study, the functional relevance of differentially expressed miRNAs was determined based on target prediction and subsequent pathways analysis in which individual or cluster of miRNAs are involved in. Comparative analysis of ANCs vs APCs revealed down regulation of miR-2285, miR-451, miR-132, miR-486 and miR-874 in EVs isolated from follicular fluid of ANCs. In-silico analysis revealed the involvement of those miRNAs in various pathways including TGF-beta signaling pathway (Fig. 5), which are known to be involved in oocyte and embryonic development. Knockdown of TGF-β pathway causes embryonic lethality in mouse^43^. Previously, upregulation of bta-miR-451 was documented in large and healthy follicles compared to small follicles in bovine^44^. Moreover, in C. elegans, TGF-β and Insulin signaling is believed to regulate the reproductive aging by modulating multiple aspects of the reproductive process, including embryo integrity, oocyte fertilizability, chromosome segregation fidelity, DNA damage resistance, and oocyte and germline morphology^45^. Among the downregulated miRNA, the bta-miR-2284 and bta-miR-2285 families, which encode more than 100 mature miRNAs in the bovine genome, are reported to be expressed in a bovine immune-relevant tissues including CD14+ monocytes, mammary epithelial cells, and others^46^. Moreover, bta-miR-132 is found to be moderately correlated with lactose in lactating cows^47^. Upregulation of miR-132 in mouse granulosa cells reported to promote estradiol synthesis by targeting the cAMP signaling pathway through translational repression of Nurr1 gene^48^. In human follicular fluid, the exosomal miRNAs; miR-132, miR-212, and miR-214 coordinately targeted Phosphatase and Tensin Homologue expression (PTEN) and have been documented to involve triggering meiosis resumption^29^. In addition, the expression of miR-485-5p was reported to be controlled by high glucose levels in humans^38^. The same study showed that overexpression of miR-486-5p induced a premature senescence-like phenotype by inhibiting proliferation of hAT-MSCs and adipogenic and osteogenic differentiation, whereas the reverse was observed by inhibition of miR-486-5p^38^.

Massive downregulation of EV-coupled miRNAs were found during comparison of ANCs versus heifers (Table 1). In that comparison, 25 out of 37 differentially expressed miRNAs were suppressed in ANCs, supporting the notion that metabolic stress impairs the release of EV-coupled miRNAs into extracellular space. One of those miRNAs was miR-21, which is a well-studied miRNA known to be involved in folliculogenesis. Upregulation of miRNA-21 in mice was found to be associated with increased cell survival and promotion of ovulation^49^. A linear increase of miR-21 was also found to be involved on controlling maternal-to-embryonic transition and early development^50^. Therefore, the downregulation of EV-coupled miR-21 in the present study due to metabolic stress may implicate the corresponding reduced expression in the follicular cells, which may impair follicular development. Similarly, elevated expression of miR-20b in bovine cumulus cells increased oocyte maturation and progesterone synthesis by targeting INHBA, MAPK1, PTGS2, PTX3, and EGFR^51^.

Comparative analysis of EV-coupled miRNAs in ANCs versus TCs at 5–6 weeks postpartum could reveal potential miRNAs as predictors of cows that will maintain negative energy balance or those that will recover in the later stages of lactation at weeks 9–10 postpartum. In that comparison, all four differentially expressed miRNAs, miR-132, bta-miR-34b and bta-miR-34c from the mir-34 family, and bta-miR-449a were found to be suppressed in cows that remained under metabolic stress throughout the experimental period. Interestingly, miR-132 and miR-34 were highly abundant in the follicular fluid of cows that under-went ovarian hyperstimulation compared to unstimulated ones^52^. This may indicate that metabolically stressed postpartum cows exhibited delayed resumption of ovarian cyclicity that may have been associated with disturbed serum metabolite profiles^53^. Therefore, the candidate miRNAs can be potential indicators of persistent or transient occurrence of metabolic stress in postpartum cows. Future research need to be done to validate this notion in an independent population and further functional studies need to be done to establish these candidate miRNAs as predictors of persistent or transient metabolic stress in dairy cows.

Irrespective of the metabolic status, comparative analysis of APCs and heifers enabled us to identify circulatory miRNAs associated with lactation physiology. Cows under positive energy balance postpartum showed induction of 31 miRNAs compared to unstressed control heifers as indicated in Table 2. Seven miRNAs were differentially expressed in common between APC and ANCs when both compared to heifers. Out of these bta-miR-184 and bta-miR-205 were downregulated and the other five miRNAs (i.e., bta-miR-26a, bta-miR-423-5p, bta-miR-6518, bta-miR-6520, and bta-miR-92b) were upregulated in both ANCs and APCs compared to heifers. This could be the result of the interaction of metabolism and lactation induced change on the abundance of EV- coupled miRNAs. This could be associated with the nutritional change after parturition in the cows and may have a short-term effect on the follicular microenvironment. Previously high lactation performance has been reported to be associated with high abundance of miRNA-29 expression in dairy cow mammary epithelial cells^54^. Based on this fact and the results of the present study, the lactation physiology of the animals may have a profound effect on the transcriptome of follicular cells and subsequent release of EV-coupled miRNAs into extracellular space. However, further studies need to be done to elucidate the effect of lactation in follicular cells transcriptome and its association with oocyte physiology.

## Conclusion

The present study revealed that negative energy balance in postpartum cows is mainly associated with downregulation of EV-coupled miRNAs in follicular fluid, whereas upregulation of EV-coupled miRNAs was associated with positive energy balance in dairy cows. Moreover, lactation induced changes in follicular fluid EV-coupled miRNA profiles in dairy cows regardless of energy status. The divergent expression of EV-coupled miRNAs in follicular fluid of metabolically stressed cows could partly explain the reduced fertility in high yielding dairy cows. Resolving the array of EV-coupled miRNAs in follicular fluid of metabolically divergent cows will undoubtedly extend our understanding on the molecular mechanisms associated with the effect of metabolic stress in ovarian functionality. Further validation of the candidate markers from this study in different population and other stress relevant experimental setup could lead to the development of markers which can be used as foundation to enhance reproductive function or treat ovarian dysregulation.

## Material and Methods

### Assessment of body weight and overall energy balance

Experimental animal handling was done in accordance with the 2015 German law of animal protection (TierSchG & TierSchVersV). Experimental protocols were approved by the state office for Nature, Environment and Consumer protection of North Rhine-Westphalia, Germany (Landesamt für Natur, Umwelt und Verbraucherschutz Nordrhein-Westfalen, Deutschland) with respect to blood sample collections by licensing number 84-02.04.2015.A139 and with respect to Ovum Pick Up (OPU) procedure by licensing number 84-02.04.2014.A500. All animals were kept in the dairy farm of the Frankenforst teaching and research station at the University of Bonn. Holstein Friesian cows (n = 30) and heifers (n = 8) were used and kept under conditions that enabled feed-intake, milk yield, and body weight data collection from individual cows. The body weight, milk yield, and dry matter intake of individual cows were measured daily for 15 weeks postpartum. Moreover, individual cow dry matter and concentrate intake was measured daily. Following that, the overall energy balance of individual cows was calculated on weekly basis using the following equation: Energy balance (EB) (MJ NEL/d) = Energy intake (EI) − Energy consumption (EC). Whereby: Energy intake (EI): Dry matter concentration of PMR (partial mixed ration) × Intake PMR (kg fresh matter) × energy concentration of PMR (MJ NEL/kg DM) plus concentrate intake (kg) × energy concentration of concentrates (MJ NEL/kg) and Energy consumption (EC): minus energy for maintenance: (0.293 × body weight (BW) 0,75) minus milk energy: (0.39 × fat% + 0.24 × protein% + 0.17 × lactose% + 0.07) × kg milk).

### Blood serum metabolite analysis

Blood samples were collected from individual postpartum cows (n = 30) at weeks 5, 6, 7, 8, 9 and 10 post-calving and in parallel to ovum pick-up time. Similarly, blood samples were collected from heifers (n = 8) during ovum pick-up sessions for five consecutive weeks. Approximately, 20 ml of blood were collected from all postpartum cows and heifers at each collection time pointinto additive carrier serum separation tubes (Sarstedt AG & Co. KG, Nümbrecht, Germany). Blood samples were cooled for 30 min at 4 °C and centrifuged at 2500 × g for 10 min to separate blood serum from the blood cells and clotting factors. Serum samples from individual postpartum cow and heifers were subjected to metabolite analysis, including analysis of nonesterified fatty acids (NEFA) and beta-hydroxybutyrate (β-OHB), in order to determine energy status of experimental animals as previously published in^55^. Briefly, NEFA concentration was quantified using series NEFA-HR (Wako Chemicals GmbH, Neuss, Germany) according to the manufacturer’s protocol. Seven micro liter of the sample calibrator was added into 300 µl of blood serum and incubated at 37 °C for 7.5 minutes. The concentration of NEFA was measured with a main wavelength of 546 nm and sub-wavelength of 660 nm. Similarly, the concentration of β-OHB was quantified using Ranbut D-3-hydroxybutrate with the BHBA kit (# RB1008) (Randox laboratory limited, United Kingdom) according to the manufacturer’s protocol.

### *In vivo* follicular fluid collection using Ovum pick-up

Collection of follicular fluid (FF) from postpartum cows (n = 30) and heifers (n = 8) was performed using an ovum pick-up (OPU) technique from large follicles with diameters >10 mm. Follicular fluid collection was done from postpartum cows at weeks 5, 6, 7, 8, 9 and 10 postpartum. Heifer FF collection was performed for five consecutive weeks. Oocytes were picked under the microscope prior to centrifugation. Follicular fluid was centrifuged at 500 g for 5 min at room temperature to remove cell debris and pellet granulosa cells. Follicular fluid samples were then transported in liquid nitrogen and stored at −80 °c until further analysis.

### Isolation of extracellular vesicles from follicular fluid using ultracentrifugation

Prior to pooling FF samples, the metabolic statuses of individual postpartum cows were assessed using three criteria: blood serum metabolite analysis (5–10 weeks), body weight curves (1–15 weeks), and overall energy balance based on dry matter intake (1–15 weeks). Based on these three criteria’s, animals were grouped in to three phenotypes namely: Transient cows (TCs) (n = 20), Always Negative cows (ANCs) (n = 5) and Always positive cows (APCs) (n = 5) (Fig. 2). Follicular fluid samples from cystic ovaries and blood contamination are discarded from the analysis. Follicular fluid samples from TCs at week 5 and 6 (n = 11) and at week 9 and 10 (n = 7) were separately pooled to create three biological replicates. Follicular fluid collected from ANCs (n = 2) and APCs (n = 2) at week 5 and 6 were pooled to generate two biological replicate per group of cows. Extracellular vesicles were isolated from a pool of 2 ml FF using differential centrifugation followed by ultracentrifugation fitted with a SW 55 Ti Rotor (Beckman Coulter, Germany). Briefly, FF was diluted with an equal amount of PBS-CMF (2 ml). Follicular fluid was centrifuged at 500 × g for 10 minutes to remove cells debris followed by another centrifugation at 6000 × g for 20 minutes to remove apoptotic bodies. The supernatant was transferred to a new tube and centrifuged at 18,000 × g for 30 minutes to remove microvesicles. The supernatant containing extracellular vesicles was transferred to a new 5 ml polypropylene centrifuge tube (Beckman Coulter, Krefeld, Germany) and centrifuged at 120,000 × g for 70 minutes to pellet EVs. The EV pellets were re-suspended with 5 ml of PBS and centrifuged at 120,000 × g for 90 minutes. Finally, EV pellets were resuspended in final volumes of 1 ml in PBS and stored in −80 °C until further analysis.

### Nanoparticle tracking and electron microscopy analysis of extracellular vesicles

Nanoparticle tracking analysis was performed to determine the concentration and size distribution of isolated extracellular vesicles using Nano Sight NS300 following the manufacturer’s protocol (Malvern Instruments, Malvern, UK). Briefly, 25 μl of EV pellet was mixed with 1 ml of PBS-CMF and assembled for five video recordings. Data was analyzed using NTA version 3.2 and the mean, mode, standard deviation, and concentration of particles were obtained. Furthermore, EVs were morphologically characterized using an electron microscope (Ziess EM109, Carl Zeiss). Briefly, 20 μl drops of purified EVs on parafilm were covered by Formvar-carbon-coated grids. Five minutes later, the Formvar-carbon-coated grids were washed using drops of PBS-CMF and incubated in 30 μl of 2% uranyl acetate. Grids were washed with drops of PBS-CMF and examined under electron microscope.

### Western blot analysis

Detection of two EVs protein markers CD63^23,56^ and Alix^24,56^ was performed by western blotting. Extraction of EV protein was performed using radioimmuno precipitation assay buffer (RIPA) according to manufacturer’s instruction (Sigma-Aldrich, Germany). Briefly, isolated EV pellets were resuspended in 100 µl of RIPA buffer and vortexed for 15 seconds. The re-suspended EVs were placed at room temperature for 5 minutes to allow complete lysis. Prior to loading for western blotting the concentration of extracted protein samples were measured using NanoDrop 8000 spectrophotometer (NanoDrop Technologies, Germany). About 30 µg of extracted protein was loaded on to a SDS gradient gel, electrophoresed, and blotted to nitrocellulose membrane using Biorad Pac 3000 transblot SD semi-DRY transfer cell (BioRad, USA). Furthermore, membrane was incubated in 10 ml blocking buffer for 1 hr at room temperature followed by incubation overnight with primary antibody at 4 °C. For this Primary antibodies of CD63 and Alix (System biosciences, Palo Alto, Canada) were diluted with blocking solution in a ratio of 1:300. Afterwards, the membrane was incubated for 1 hr with secondary antibody of Goat Anti-Rabbit HRP (System biosciences, Palo Alto, Canada) with a dilution of 1:8000 in blocking solution. The membrane was further incubated for 5 minutes using equal amount of peroxide and luminol/enhancer solution (BioRAD, USA). The bands were visualized using the ChemiDoc™ XRS + system (Bio-Rad Laboratories GmbH, USA).

### Isolation of total RNA including miRNAs

Isolation of EV total RNA enriched for miRNA was done using an exosomal RNA isolation kit (Norgen, Canada) according to the manufacturer’s protocol. Briefly, isolated EVs suspended in 400 µl PBS-CMF were lysed with 600 µl of lysis buffer and 75 µl of lysis additive and incubated for 10 minutes at room temperature. After incubation, 500 µl of absolute ethanol was added to the solution and transferred to a mini spin column followed by centrifugation at 3,300 × g and washing steps. Total RNA was eluted in 32 µl of elution solution. Prior to downstream applications, the concentration and integrity of the total RNA were assessed using NanoDrop 8000 spectrophotometer (NanoDrop Technologies, DE) and Agilent 2100 Bioanalyzer (Agilent Technologies, CA), respectively.

### Next generation sequencing and data analysis

Illumina-based next generation sequencing (NGS) of small RNA and miRNA was performed (Qiagen, Germany). For each sample, a total concentration of 160 ng with the maximum input volume of 5 µl was processed using the QIAseq miRNA Library Prep kit according to the manufacturer’s protocol. Sequencing was performed on an Illumina NextSeq. 500 sequencing instrument (Qiagen, Germany). The quality of raw FASTQ file was assessed using FastQC version 0.11.4 (http://www.bioinformatics.babraham.ac.uk/projects/fastqc/). Data analysis was performed according to the XploreRNA pipeline (Qiagen, Germany). Briefly, adaptors were trimmed from the raw sequences using Cutadapt (1.11) and mapped against the indexed bovine reference genome (UMD3.1) using Bowtie2 (2.2.2) software with a criterion of one mismatch in the first 32 bases of the reads without insertion or deletion. Sequence reads, which were aligned to the bovine reference genome, were then used for annotation against bovine precursor miRNAs and matured miRNAs in the miRbase database, release 20. Normalization of raw expression data was done using the trimmed mean of M-values (TMM method of normalization), which is based on log-fold and absolute gene-wise changes in expression levels between samples. Differential expression analysis was performed on the TMM normalized expression values using EdgeR statistical software package (Bioconductor, http://www.bioconductor.org/). A miRNA with a log_2_ fold change of 1 ≥ log_2_ ≤ −1p-value < 0.05 and false discovery rate of ≤0.25 was considered as statistically significant. The potential pathways enriched by differentially expressed miRNAs were analyzed using DIANA-miRPath v3 (http://snf-515788.vm.okeanos.grnet.gr/). Moreover, miRNA-gene network analysis was performed using ingenuity pathway analysis (https://www.qiagenbioinformatics.com/products/ingenuity-pathway-analysis/).

## Supplementary information

Supplementary Figures

Suppelmentary Table 1

Suppelmentary Table 2

Suppelmentary Table 3

Suppelmentary Table 4

## Data Availability

The authors confirm that all data underlying the findings are fully available. Sequence data files are available in GEO database with Accession Number: GSE129367. To review GEO accession GSE129367: Go to https://www.ncbi.nlm.nih.gov/geo/query/acc.cgi?acc=GSE129367 Enter token uxypugogvrchxqn into the box.
