# Supplementary material for: Extracellular vesicle-coupled miRNA profiles in follicular fluid of cows with divergent post-calving metabolic status
Source: Sci Rep. 2019 Sep 6;9:12851. doi: 10.1038/s41598-019-49029-9 (PMC6731312; doi:10.1038/s41598-019-49029-9)
Supplement: Supplementary file 1 — Supplementary Figures [file 41598_2019_49029_MOESM1_ESM.pdf]

Extracellular vesicle-coupled miRNA profiles in follicular fluid of cows with divergent post-calving metabolic status

Tsige Hailay, Michael Hoelker, Mikhael Poirier, Samuel Gebremedhn, Franca Rings, Mohammed Saeed-Zidane, Dessie Salilew-Wondim, Christina Dauben, Ernst Tholen, Christiane Neuhoff, Karl Schellander and Dawit Tesfaye*

List of supplementary figures

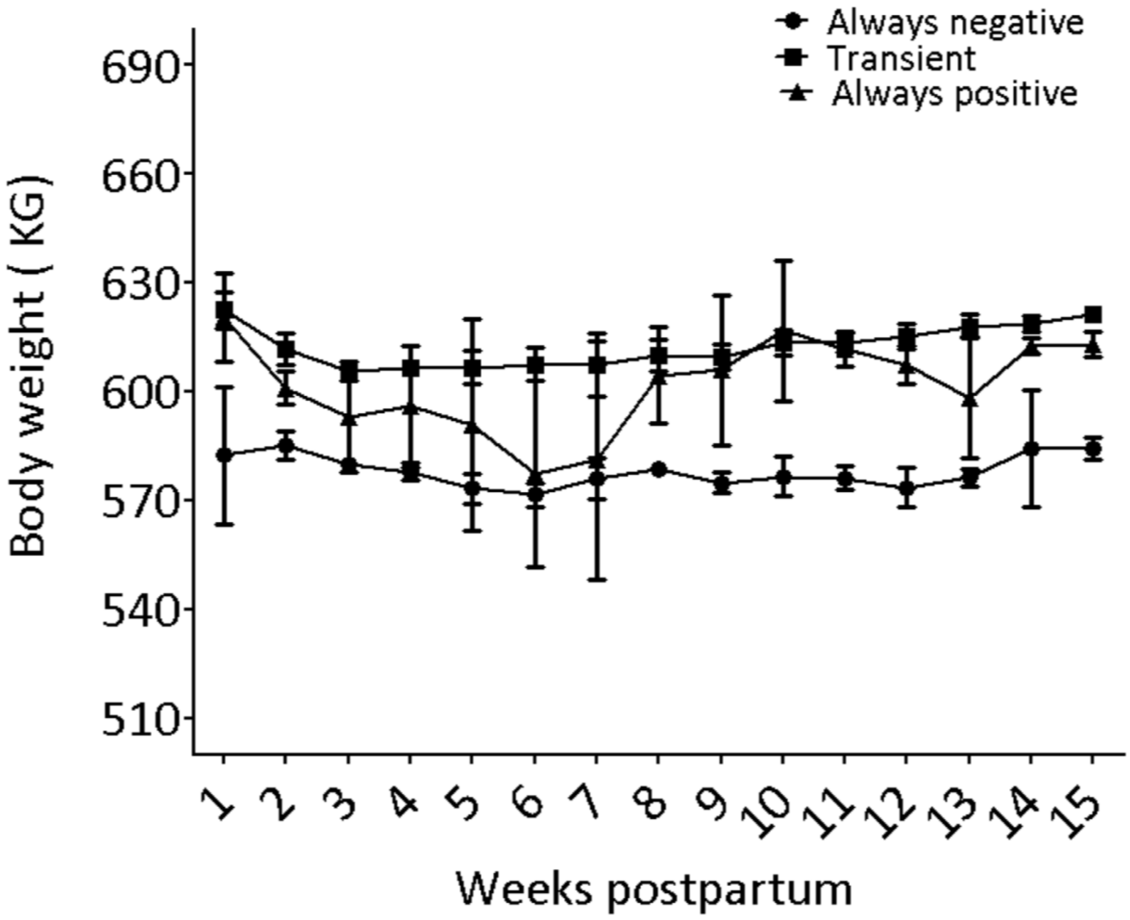

Supplementary fig. S1: Body weight curve of postpartum cows of different metabolic status.

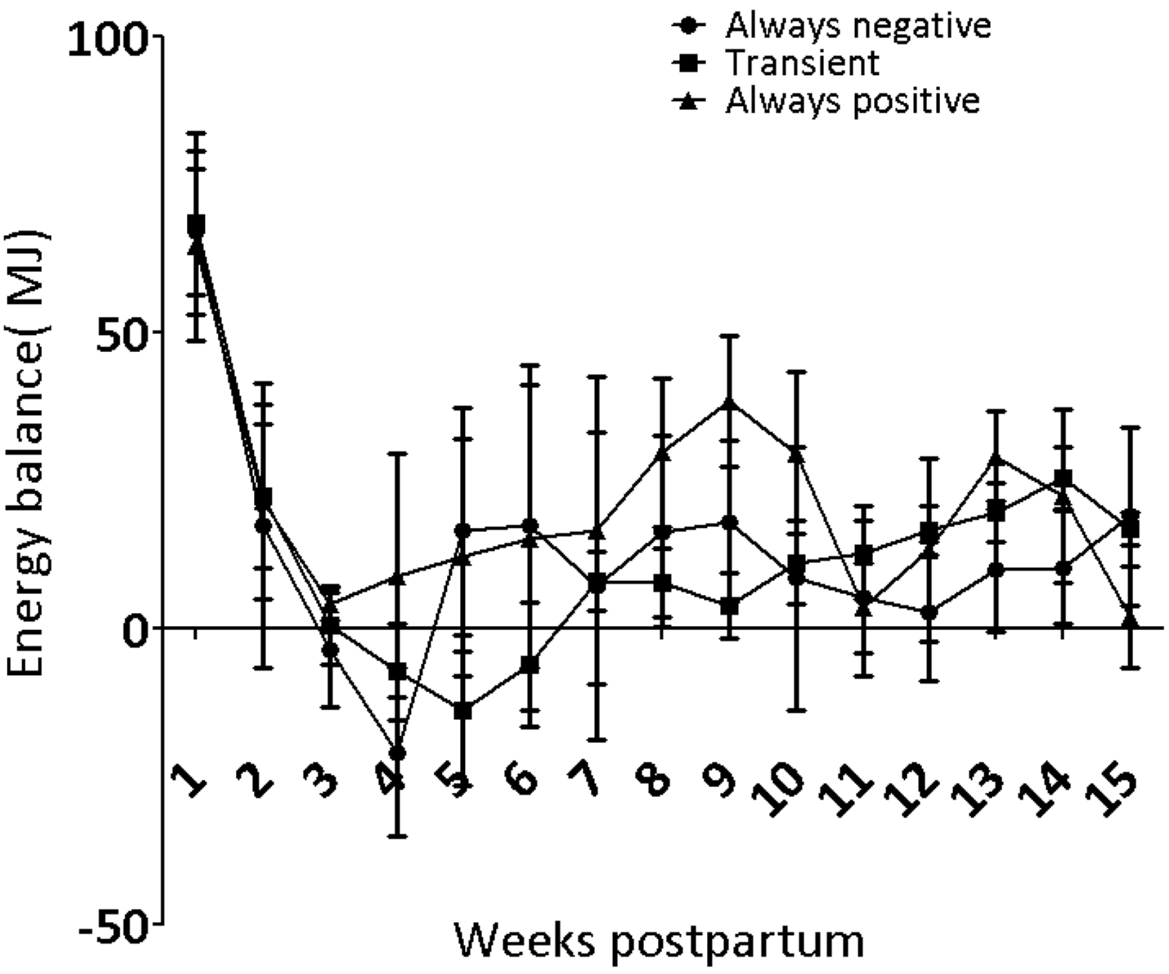

Supplementary fig. S2: Over energy balance curve of postpartum cows based on their dry matter intake.

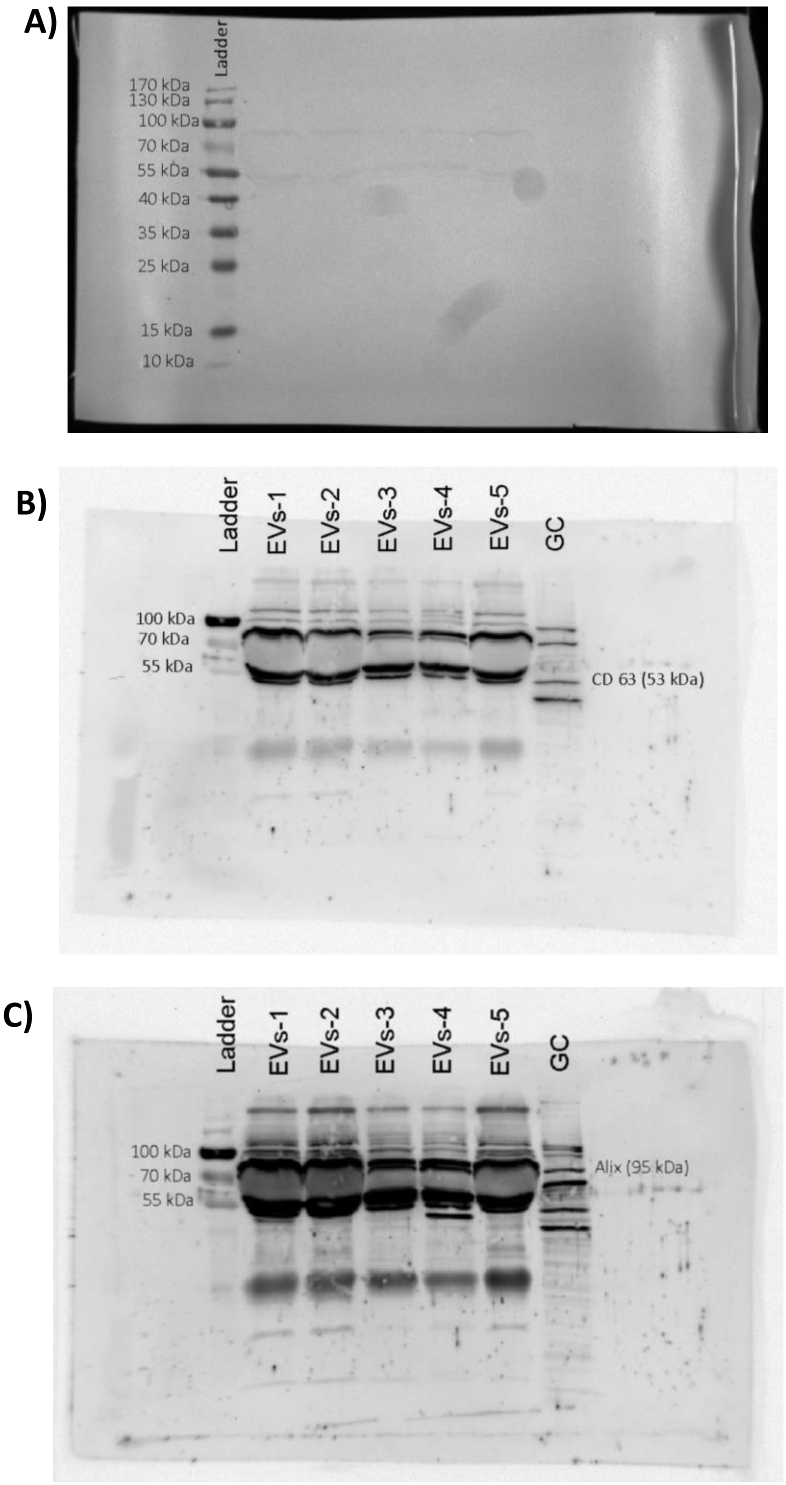

Supplementary fig. S3: Detection of protein markers using western blot in EVs recovered from different metabolic status cows: A) Ladder used for western blotting of protein detection for CD63 and Alix. **B)** Western blot showing CD63 protein detection in EVs recovered from follicular fluid of different metabolic status cows. **C)** Western blot showing the detection of Alix protein in EVs recovered from follicular fluid of different metabolic status cows. The numbers represent different sources EVs 1: EVs of TCs (weeks 5 - 6), 2: EVs of TCs (weeks 9 - 10), 3: EVs of ANCs, 4: EVs of APCs, 5: EVs of heifers and GC: Granulosa cells used as a control.

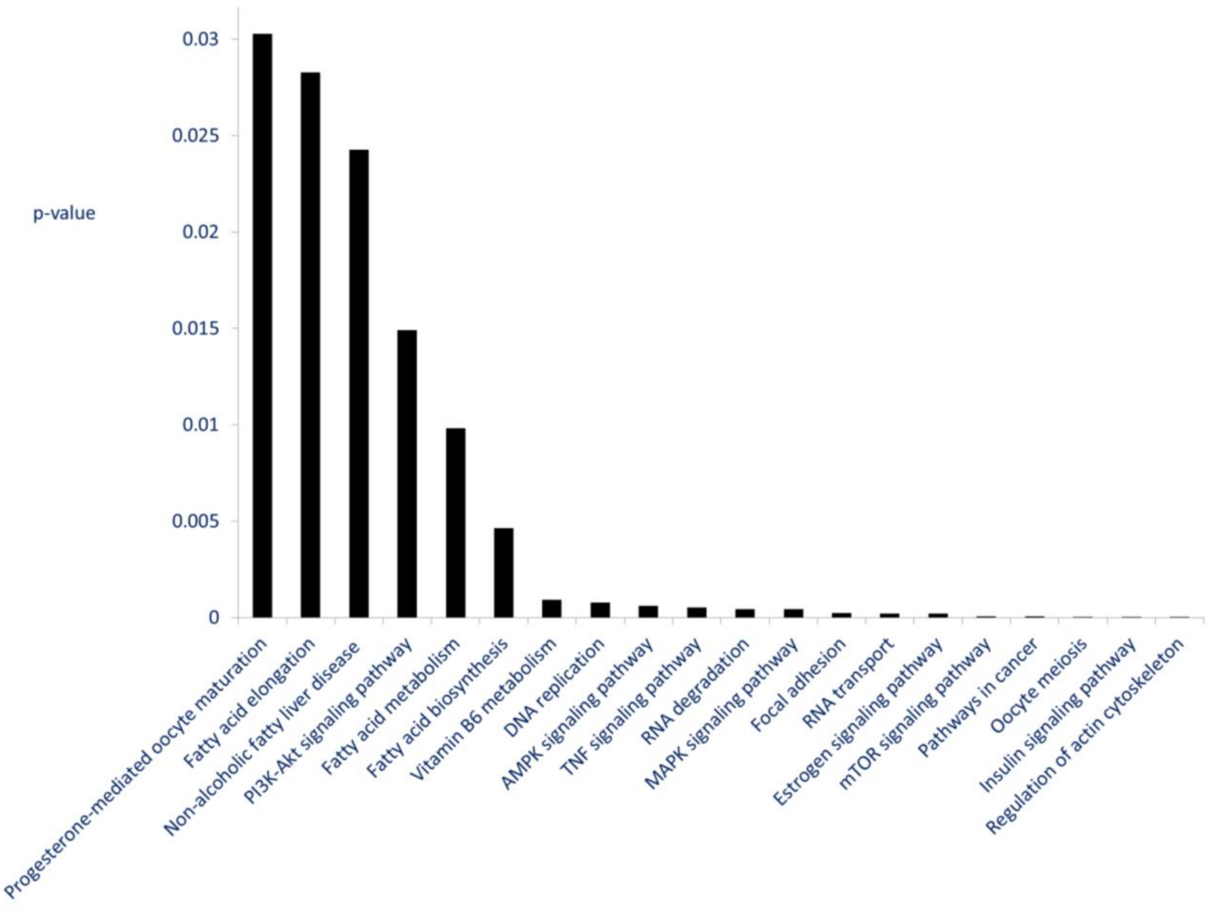

Supplementary fig. S4: Target prediction analysis of downregulated miRNA in always negative cows vs heifers.

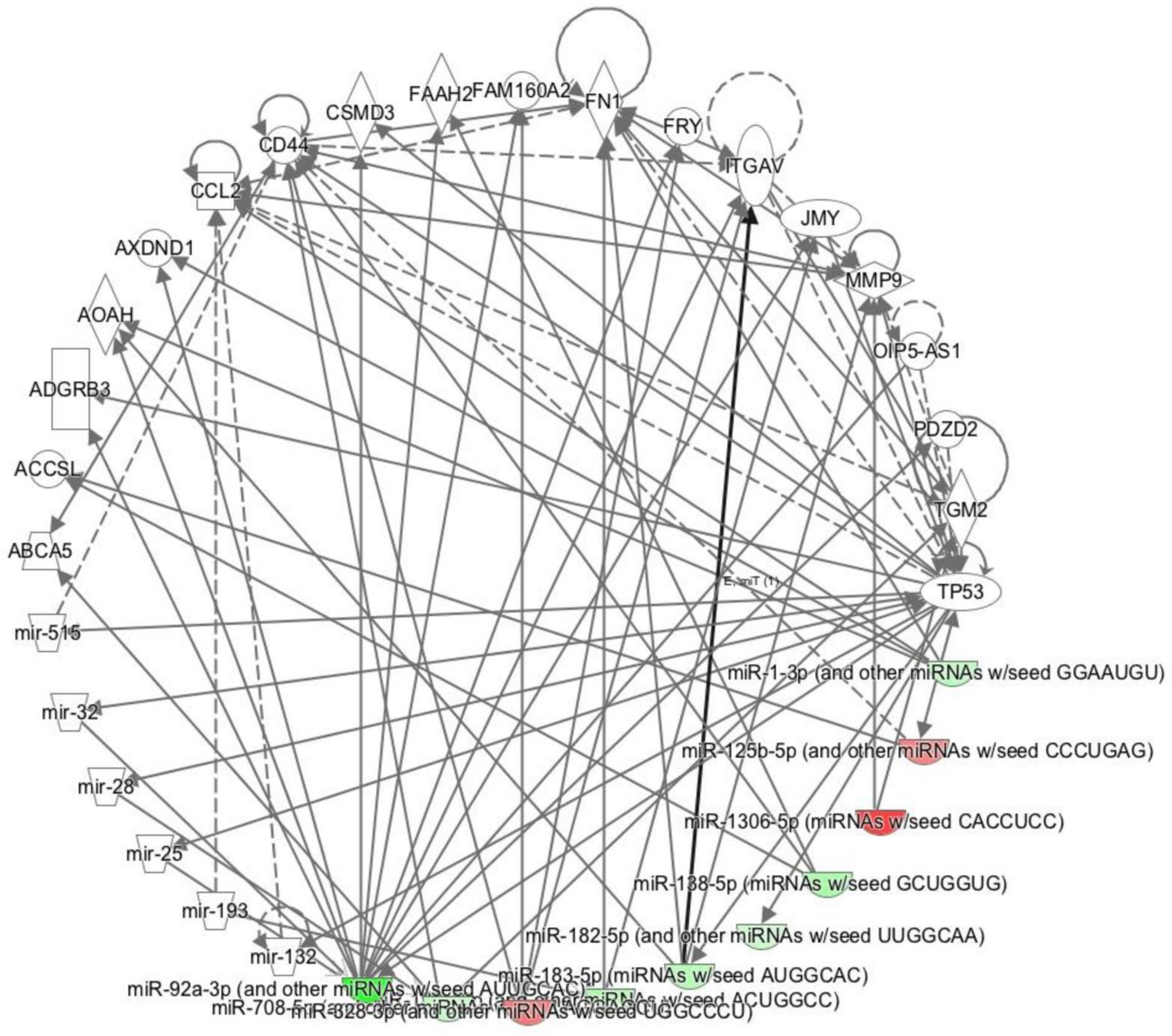

Supplementary fig. S5: miRNA-gene network of differentially expressed miRNAs in always negative cows vs heifer

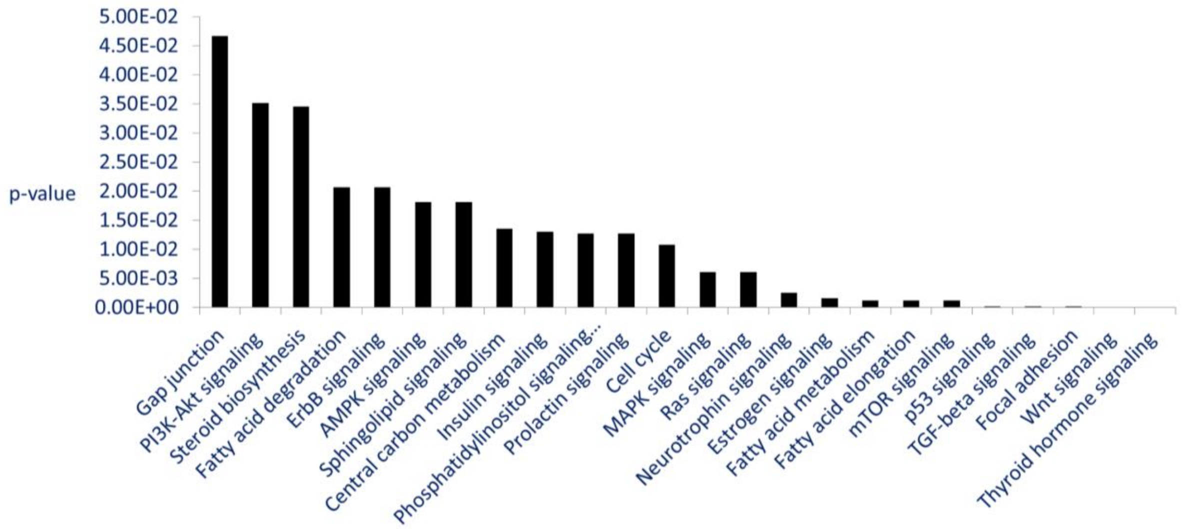

Supplementary fig. S6: Target prediction analysis of upregulated miRNAs in Always positive cows vs heifers upregulated.
